# How sensitive are protein hydration shells to electrolyte concentration and protein composition?

**DOI:** 10.1002/pro.5241

**Published:** 2024-12-14

**Authors:** H. Geraili Daronkola, Bashar Moussa, Óscar Millet, Oktavian Krenczyk, Gabriel Ortega‐Quintanilla, Poul B. Petersen, Ana Vila Verde

**Affiliations:** ^1^ Faculty of Physics University of Duisburg‐Essen Duisburg Germany; ^2^ Faculty of Chemistry and Biochemistry Ruhr‐University Bochum Bochum Germany; ^3^ CIC bioGUNE, Asociación Centro de Investigación Cooperativa en Biociencias Derio Bizkaia Spain; ^4^ Ikerbasque, Basque Foundation for Science Bilbao Spain

## Abstract

Proteins of obligate halophilic organisms have an unusually high number of acidic amino acids, thought to enable them to function in multimolar KCl environments. Clarifying the molecular scale mechanisms by which this occurs is relevant for biotechnology, to enable enzymatic synthesis of economically important small molecules in salty environments and other environments with low water activity. Previous studies have suggested that acidic amino acids are necessary at high salt concentration to keep the proteins hydrated by competing with the ions in solution for available water (the “solvent‐only” model). We use a combination of solvation shell spectroscopy and molecular dynamics simulations for in total 13 proteins, at high and low KCl concentration, to investigate this scenario. We show that the solvation shells of halophilic and mesophilic proteins of widely different amino acid compositions, net charges, sizes, and structure respond similarly, in terms of composition and of hydrogen bond network, to changes in KCl concentration. The results do not support the solvent‐only model, and point to other mechanisms behind the acidity of halophilic proteins. Excess acidic amino acids may ensure protein solubility by the combined effects of having particularly favorable electrostatic interactions with the solvent, ensuring very short range protein–protein repulsion, and having smaller hydrophobic solvent accessible surface area than other charged amino acids. Also possible is that highly acidic proteins are well‐tolerated—but not necessarily indispensable—in terms of stability and solubility.

## 1 INTRODUCTION

Halophilic microorganisms, also called halophiles, are extremophiles that thrive in environments with up to multimolar concentrations of NaCl (Deole et al., 2013; Graziano & Merlino, 2014; Gunde‐Cimerman et al., 2000; Lanyi, 1974; van der Wielen et al., 2005). The main challenge posed by this environment to typical (here called mesophilic) organisms is the high osmotic pressure difference which arises because their cytoplasm contains only ≈0.15 M of osmolytes. Mesophilic organisms thus desiccate and die in environments with high electrolyte concentrations. By accumulating osmolytes in their cytoplasm up to concentrations similar to their environment, halophilic organisms overcome this problem and not only survive but thrive in these environments. For halophiles living in media where the external NaCl concentration is above ≈1 M, the typical osmolyte is the inorganic salt KCl rather than small organic molecules; halophiles with cytoplasmic concentrations of up to 4 M of KCl exist (Brininger et al., 2018; Deole et al., 2013; Elevi Bardavid & Oren, 2012; Lanyi, 1974; Paul et al., 2008; Peters et al., 2023).

The cytoplasmic proteins of this type of halophilic organism—here called halophilic proteins—thus function under salt concentrations thought inhospitable for mesophilic proteins (Dumetz et al., 2007; Wright et al., 2002). In contrast with mesophilic proteins, halophilic proteins often unfold or become inactive at low KCl concentration (Graziano & Merlino, 2014; Kennedy et al., 2001; Lanyi, 1974; Paul et al., 2008; Siglioccolo et al., 2011). The different response of these two types of protein clearly must stem from their different amino acid composition: halophilic proteins are rich in acidic and small+polar amino acids, at the expense of basic and large+ apolar amino acids. However, the mechanisms providing the evolutionary driving force for these differences remain under discussion, as briefly summarized below (Graziano & Merlino, 2014; Kennedy et al., 2001; Madern & Zaccai, 2004; Paul et al., 2008). This understanding is important to design enzymes for cell‐free biotechnological processes that can take place in saline water (e.g., to produce biofuel, bioplastics, chiral molecules, high‐value molecules like isoprenoids, molecules with unusual functional groups like fluorinated carbons), thus preserving fresh‐water resources. (Bangaru et al., 2022; Bornscheuer et al., 2012; DasSarma & DasSarma, 2015; Horn et al., 2012; Meghwanshi et al., 2020; Rossino et al., 2022) Because they function in environments with low water activity, halophilic proteins might reveal modifications that enhance the function of enzymes in other low water activity environments such as those experienced by PETases (which degrade PET plastics) (Katsimpouras & Stephanopoulos, 2021) and other hydrolases (De Lourdes Moreno et al., 2013; Garg et al., 2016). Moreover, because proteomic and genomic analyses suggest that salt and pH adaptation share some mechanisms, understanding the mechanistic origin of halophilicity might assist in developing enzymes for biotechnological applications at different pH levels (Amangeldina et al., 2024).

Changes in protein thermodynamic stability, solubility, and activity as a function of KCl concentration necessarily stem from changes in magnitude of the hydrophobic effect—the force driving hydrophobic molecules to aggregate in aqueous solution beyond their direct intermolecular interactions—and of electrostatic screening with increasing salt concentration, as well as from the contribution from specific ion–protein interactions (ion‐binding). On the one hand, the surface tension of KCl (and NaCl) solutions increases with ionic strength (Date & Dominy, 2013; Pegram et al., 2007; Thomas & Elcock, 2007; Zangi et al., 2007), which means that the free energy cost of exposing hydrophobic groups to the solvent increases with KCl concentration (Clever, 2013). On the other hand, electrostatic screening increases with ionic strength.

Continuum models confirm that both the hydrophobic effect and electrostatics are necessary to quantitatively understand changes in protein stability as a function of salt concentration (Date & Dominy, 2013). It follows that both effects will also be important to understand protein activity, because protein activity is surrogate to its conformational stability: only certain conformers have the right structure to enable a given enzymatic or other function (Ortega et al., 2011; Wright et al., 2002). That said, the salt dependence of protein activity is often different from that of thermodynamic stability (Tehei et al., 2001; Wright et al., 2002), partially because activity also depends on protein dynamics (flexibility) (Luk et al., 2015; Wang et al., 2011), and partially because is affected by changes in the binding affinity of substrates (Wright et al., 2002). Both the hydrophobic effect and electrostatics regulate the binding affinity of substrates; however, KCl impact on protein dynamics appears to be largely via electrostatic effects (Geraili Daronkola et al., 2024), contrary to expectations (Mevarech et al., 2000; Tehei et al., 2001).

The impact of KCl on protein solubility is also not straightforward to understand. At very low salt concentrations, experiments have shown that protein–protein interactions can be attractive or repulsive, primarily depending on the net charge and on the charge distribution at the protein surface (Dumetz et al., 2007). Changes in KCl concentration strongly impact the solubility of proteins in solution as measured by the osmotic second virial coefficient ($b_{2}$) (Guo et al., 1999) for salt concentrations up to ≈0.5 M predominantly due to electrostatic screening and, for some electrolyte ions, ion‐binding (Dumetz et al., 2007). Beyond this threshold, however, electrostatic screening is largely complete, so one would expect that the hydrophobic effect increase with salt concentration would lead to protein aggregation and precipitation. After all, salt has a dramatic effect on the solubility of hydrophobic compounds, for example, the solubility of methane at 4 M KCl is only ≈1/3 of that in water (Clever, 2013). Somewhat surprisingly, however, $b_{2}$ varies little with increasing KCl (and NaCl) (Tessier & Lenhoff, 2003) concentration for some proteins, whereas it decreases (i.e., protein–protein interactions become more attractive) for others (Dumetz et al., 2007). Relatedly, the x‐ray scattering intensity (which reflects solute–solute interactions) of concentrated solutions of the hydrophobic amino acid leucine in solution changes little as a function of NaCl concentration (Song et al., 2022). These different responses to increases in KCl concentration beyond ≈0.5 M are possibly because of a different balance between cost/gain of desolvating polar groups vs. apolar groups as a function of salt concentrations for different protein compositions (Dumetz et al., 2007).

Modeling suggests that the abundance of acidic amino acids, and thus a net negative charge, in halophilic proteins might reduce aggregation and thus enhance solubility at high salt concentration (Elcock & McCammon, 1998; Graziano & Merlino, 2014). Beyond this possible mechanism, it has also been proposed that the abundance of acidic amino acids in halophilic proteins leads to quantitative differences in their interaction with the solvent that explain their stability and solubility at high KCl concentrations. The ion‐solvent model claims that halophilic proteins counterbalance the larger tendency to aggregate at high salt concentrations by ensuring that their solvation layer has a composition close to the bulk of the solution, that is, by reducing the surface tension at the protein–solvent interface. In this view, the large abundance of acidic amino acids at the surface of halophilic proteins is evolutionarily driven by cooperative, stabilizing interactions with cations and water that predominate in the folded, but not the unfolded, conformation (Pundak et al., 1981). Despite the expected repulsion, the acidic amino acids should stabilize the folded protein structure by forming cooperative ion‐water networks (Madern et al., 2000; Zaccai et al., 1989), which according to this view manifest as ordered protein hydration shells with very slow dynamics (Graziano & Merlino, 2014). This model was based on the observation, using neutron scattering and ultracentrifugation, that some folded halophilic proteins show significant binding to water, to NaCl or to KCl (Calmettes et al., 1987; Pundak & Eisenberg, 1981); in contrast, non‐halophilic proteins show no salt binding in the native state (Madern et al., 2000). The cooperativity aspect of the stabilization is important in this model, because carboxyl groups are strongly hydrated both in the folded and in the unfolded forms of a peptide (Kuntz, 1971a); only if binding of water and ions to the folded structure is enhanced, via a cooperative effect, relative to the unfolded structure would it result in stabilization of the folded structure. Our recent simulation results have found some evidence for cooperative interactions involving carboxylates, cations and water (Geraili Daronkola & Vila Verde, 2023), but also found that they exist—likely less frequently—in unfolded protein conformations as well, and do not require rigid conformations of the acidic side chains. We also found no evidence (Geraili Daronkola & Vila Verde, 2021) for the pronounced slowdown of water dynamics postulated by this model based on the interpretation of quasielastic neutron spectroscopy observations (Jasnin et al., 2010), in agreement with ^17^O magnetic relaxation experiments that found that the rotational dynamics of solvation shells was similar for halophilic and mesophilic proteins (Qvist et al., 2012).

Another proposed mechanism for the role of acidic amino acids in protein halophilicity is the solvent‐only model. The model claims that an abundance in acidic amino acids ensures that halophilic proteins remain hydrated at high KCl concentrations—that is, in environments with low water activity—by competing (and not cooperating, as in the ion‐solvent model) with the ions in solution for available water (Deole et al., 2013; Frolow et al., 1996). In this scenario, a robust hydration shell would both prevent proteins from aggregating and would keep them functional; acidic amino acids are preferable over others because they have the highest water‐binding capacity of all natural amino acids (Kuntz, 1971b). Our prior simulation work (Geraili Daronkola & Vila Verde, 2021), however, found very little change in the level of hydration of both mesophilic and halophilic folded proteins when increasing the KCl concentration from 0.15 mol kg^−1^ to 2 mol kg^−1^, despite the fact that the protein solvation shells are enriched in counterions relative to the solvent in the bulk, and that the local potassium and chloride concentration near the proteins depends on the bulk one (Geraili Daronkola & Vila Verde, 2021).

The above summary makes clear that directly probing the structure and composition of protein solvation shells as a function of salt concentrations would help discern between the different models that have been proposed to explain the role of acidic amino acids in determining the ability of proteins to remain stable, folded and functional in aqueous solutions at high salt concentrations. To date, however, there are no experimental studies that give direct insight into the solvation shells of halophilic and mesophilic proteins using non‐perturbative methods. Here, we address this knowledge gap by combining IR Solvation Shell Spectroscopy (IR‐SSS) and molecular dynamics simulations with atomistic force fields. IR‐SSS is a difference spectroscopy and probes the solvation shell of solutes directly. Experiments are done on mesophilic lysozyme and protein L, as well as on a halophilic version of protein L. Simulations are used to characterize the hydrogen bonding network of 5 halophilic–mesophilic protein pairs. The combined data set of experiment and simulation, which includes proteins of different size, net charge and degrees of halophilicity, enables us to generalize our observations.

## 2 METHODS

### 2.1 Experiment

#### 2.1.1 Protein expression and characterization

As our model systems, we have studied the B1 domain of Protein L from *Finegoldia magna* (formerly *Peptostreptococcus magnus*), herein referred to as protein L. In particular, we have used the wild type (WT) mesophilic protein, and a halophilic variant (Kx5E) in which five of its surface lysines are substituted for glutamates. Extensive prior work by some of us has shown that Kx5E has many properties similar to those of natural halophilic proteins. (Tadeo et al., 2009) More specifically, while the structure and amino acid sequence of the halophilic variant is virtually identical to that of wild‐type protein L, the salt‐induced stabilization and solubility is higher and similar to that of halophilic proteins. The resulting amino acid sequences are MEEVTIKANL IFANGSTQTA EFKGTFEKAT SEAYAYADTL KKDNGEWTVD VADKGYTLNI KFAG for mesophilic protein L, and MEEVTIKANL IFANGSTQTA EFEGTFEEAT SEAYAYADTL KEDNGEWTVD VADEGYTLNI EFAG for its halophilic variant (substitutions underlined). The WT protein structure has Protein Data Bank ID 1HZ6. The structure of a protein L mutant with 6 lysine‐to‐aspartate mutations and in which 5 of the 6 mutations are the same as those in the Kx5E mutant used in the solvation shell experiments is also available in the protein data bank (pdb ID: 2KAC). This mutant serves as an excellent reference for the mutant investigated here, as prior work by some of us has demonstrated that different lysine‐to‐aspartate mutants retain a structure very similar to the wild‐type protein L (Tadeo et al., 2009). The rational for the design and the details for the production of these proteins have been already described elsewhere (Tadeo et al., 2009). Briefly, we transformed Escherichia coli BL21(DE3) (New England BioLabs) with pET16b plasmids containing these sequences between NcoI and XhoI restriction sites (GenScript), and grew the cells in LB media until an OD600 of 0.6. Then, we induced protein overexpression with the addition of 1 mM IPTG, continuing incubation at 37°C for 4 h. After harvesting the cells by centrifugation, we purified the proteins. First, we resuspended the pellet in buffer (20 mM sodium phosphate at pH 6.0), lysed the cells by sonication, and separated the supernatant by ultracentrifugation. Following this, we subjected the supernatant to a heat shock at 75°C for 5 min, allowed to recover room temperature under gentle shaking for 30 min, and centrifuged it to remove protein aggregates. Finally, we purified the supernatant by size exclusion chromatography on a HiLoad 26/600 Superdex 75 pg. column (GE Healthcare) with the same buffer. Protein L elutes at around 150 mL. We assessed protein purity by SDS‐PAGE and determined the protein concentration by measuring absorbance at 280 nm. If necessary, we performed ion‐exchange chromatography to remove nucleic acid contamination. To do this, we loaded the protein solution onto a HiTrap Q FF column (GE Healthcare) and eluted it with a linear gradient from 0 M to 1 M NaCl in 20 mM sodium phosphate at pH 6.0. Mesophilic protein L elutes at 50 mM NaCl, whereas the halophilic variant elutes at 500 mM NaCl. We then dialyzed the samples against buffer 20 mM sodium phosphate at pH 6.0, concentrated them to 0.5 mg/mL, and lyophilized them for shipment and storage. The sodium phosphate buffer is too diluted to significantly impact protein stability.

#### 2.1.2 Solvation shell spectroscopy

##### Hydrating chicken egg white lysozyme

A stock solution of pure 20 mM sodium phosphate buffer at pH 6 was prepared by dissolving sodium phosphate powders in water. 10 mL aliquots, each with KCl concentration {0.0, 0.15, 0.5, 2.0, 3.5} M were made by dissolving KCl powder (CAS‐no: 7447‐40‐7, lab-honeywell.com) with the buffer stock solution. 5 mL samples of these solutions were used to dissolve chicken egg white lysozyme powder (CAS‐no: 62971‐10G‐F; Sigma Aldrich), with the remaining 5 mL used as pure reference solution. This preparation method ensures that the reference solution at each KCl concentration is identical to that containing the protein, and should be used when possible. For protein L, a different protocol was followed because the lyophilized protein samples already contained buffer. Hydrating Protein L: Each falcon tube of the lyophilized protein L (WT or Kx5E) was rehydrated with 15 mL of an aqueous solution of the desired KCl concentration, yielding a solution with 0.5 mg/mL concentration of protein, 20 mM sodium phosphate buffer at pH 6, and KCl concentration {0, 0.15, 0.5, 2.0, 3.5} M. The KCl solutions were made in steps, by first preparing 500 mL of a stock solution with KCl concentration 3.5 M from KCl powder and water and then diluting it with water to prepare the solutions with lower concentration. Background solutions, similar to the previous ones but without protein, were prepared by using the same stock solution of 3.5 M KCl to hydrate sodium phosphate powders to obtain sodium phosphate 20 mM buffer at pH 6, preparing a second solution by hydrating phosphate powders with water to obtain the same buffer concentration, and mixing these two solutions in the volume proportions necessary to achieve KCl concentration of {0, 0.15, 0.5, 2.0, 3.5} M while keeping the buffer concentration constant. The procedures used reduce the uncertainty, $u_{\mathrm{KCl}}$, of the difference of the KCl concentration between the background and the protein solutions to $u_{\mathrm{KCl}}=\pm \left[0.002,0.01\right]$ M (the uncertainty increases for higher KCl concentrations), and the uncertainty, $u_{\text{buffer}}$, of the difference in the buffer concentration between the protein and the background solutions to $u_{\text{buffer}}=\pm 0.1$ mM. The uncertainty was calculated with the commonly used variance formula that results from error propagation neglecting correlations between the variables. All solutions containing sodium phosphate were prepared using di‐sodium hydrogen phosphate (CAS‐no: 7558‐79‐4; Sigma‐Aldrich), and sodium phosphate monobasic (CAS‐no: 7558‐80‐7; Sigma‐Aldrich) with molecular biology grade, following the same protocol (Stoll & Blanchard, 1990) used during synthesis of protein L (WT and Kx5E) to obtain 20 mM sodium phosphate buffer at pH 6. Ultrapure MilliQ water was used in all procedures. The samples were not shaken to avoid aggregation of the proteins. Circular dichroism (CD) and dynamic light scattering (DLS) spectroscopy confirm that the two variants of protein L are not denatured and do not aggregate even at higher protein concentrations than those used in IR‐SSS (see CD spectra in Figure S1 and the discussion in section S1).

##### IR‐SSS procedure

The ATR‐FTIR spectra were taken using a Thermo‐Fisher Nicolet iS50 with a temperature‐controlled Germanium ATR crystal (PIKE). The ATR‐crystal was connected to a water bath held constant at a temperature of 25°C. The ATR crystal was cleaned before each sample using the following procedure: the crystal was first cleaned using acetone and dried for 30 s, then cleaned with ultrapure water and dried for 30 s. After cleaning, a new background was taken before taking each ATR spectrum. The samples were measured starting from the lowest to the highest concentration. First, the spectrum of the pure buffer without protein was taken before the protein solution at the same salt concentration, after which the ATR crystal was cleaned again before the next protein sample. The samples were pre‐equilibrated to 25°C in the water bath and were further left for 2 min on the ATR crystal to equilibrate before measuring the spectrum. The ATR FTIR spectra were taken with a resolution of 4 cm^−1^ and averaged over 32 scans. The frequency range of the measurement was [1000, 4000] cm^−1^.

##### IR‐SSS data treatment

Solvation‐shell spectroscopy is essentially a difference spectroscopy, where the spectrum of a solution is considered as a two‐component system composed of the solute with associated solvation‐shell and the bulk solvent. The spectrum of the solute with its associated solvation‐shell (the solute‐correlated spectrum) is extracted from the total spectrum by subtracting out the bulk solvent contribution. In the subtraction, the spectrum of the bulk solvent is multiplied with a coefficient to account for the volume occupied by the solute with solvation‐shell, that is, if the solute occupies 1% then the coefficient is 0.99. The optimal coefficient is found as the largest coefficient that does not lead to negative spectral features—the least non‐negative spectrum. In the earlier studies, the optimal coefficient was first found using the MCR or SMCR mathematical routine and then adjusted slightly to account for experimental noise and baseline drifts (Daly et al., 2017; Davis et al., 2013; Perera et al., 2009; Robalo et al., 2021; Sun & Petersen, 2017). In the present study, the coefficient was found through a simple subtraction scheme, which is equivalent to the MCR/SMCR method.

Before the solvation‐shell analysis, the spectra were baseline‐corrected by subtracting a linear baseline obtained by fitting a straight line between two points where the spectral intensity is minimal. For the protein L samples, these were found as the average over [2625, 2654] cm^−1^, and over [3895.5, 3954.8] cm^−1^. The obtained linear baseline was then subtracted from the whole range of our spectra ([1000, 4000] cm^−1^). For the lysozyme data, the slightly different ranges of [2615, 2624.6] cm^−1^, and [3755.2, 4000] cm^−1^ were used for creating the baseline correction. Finally, all spectra were normalized to account for small changes in the protein concentration. This was done by normalizing to the amide II vibration by integrating the spectral intensity over the range [1500.1, 1550] cm^−1^.

### 2.2 Simulation

Simulations were performed for five pairs of halophilic–mesophilic protein pairs, referred to throughout this article by their pdb IDs: the proteins are ferredoxin (pdb ID for the halophilic version: 1DOI—pdb ID for the mesophilic version: 1FRD), protein L (2KAC‐1HZ6), beta‐lactamase (3WRT‐1ZKJ), carbonic anhydrase (4CNX‐1V9E), dihydrofolate reductase (2ITH‐2L28). The protein 2KAC differs from the Kx5E protein investigated experimentally only by an extra lysine‐to‐glutamate mutation; the wild‐type protein L is the same as used experimentally. Full simulation details are given in ref. (Geraili Daronkola & Vila Verde, 2021); here, we describe the force fields used and the production run parameters only. We used the TIP3P water model (Jorgensen et al., 1983), the AMBER ff14SB (Cornell et al., 1995; Maier et al., 2015) force field for proteins, and the K^+^ and Cl^−^ parameters of Joung and Cheatham (Joung & Cheatham, 2008) as base force fields, but modified the Lennard‐Jones parameters for interactions involving carboxylate groups (Geraili Daronkola & Vila Verde, 2021; Kashefolgheta & Vila Verde, 2017). The self‐interaction Lennard‐Jones (LJ) parameters for carboxylate oxygen atoms determined by Kashefolgheta and Vila Verde (Kashefolgheta & Vila Verde, 2017) offer a more precise approximation of acetate hydration‐ free energy in TIP3P water compared to the original AMBER parameters. Furthermore, adjustments were made to the LJ parameters governing the interaction between carboxylate groups and lysine side chains, as described in the same reference (Kashefolgheta & Vila Verde, 2017), to prevent the formation of excessively strong salt bridges. Additionally, modifications were applied to the LJ parameters governing carboxylate–potassium interactions to better reproduce the thermodynamic activity of potassium carboxylate in aqueous solution and the distances between carboxylate groups and potassium ions in the crystal structure of a protein (Geraili Daronkola & Vila Verde, 2021). To simulate the [2Fe‐2S]^2+^ ligand found in the ferredoxin proteins, we adapted the parameters developed by Carvalho et al (Carvalho et al., 2013). for a ferredoxin from *Mastigocladus laminosus* as follows: (i) The equilibrium angles were those in the crystal structure of our two ferredoxin proteins. (ii) The force constant for the Fe–S–C angle was missing in the parameter set of Carvalho et al. (Carvalho et al., 2013), so we assigned it the value recommended in that work for the desulforedoxin protein from *Desulfovibrio gigas*, which contains Fe(III) coordinated by four cysteines similarly to the desulforedoxin protein. The zinc metal center of the carbonic anhydrase proteins was simulated using ZAFF (Zinc AMBER force field) (Li et al., 2013; Peters et al., 2010) and the van der Waals (vdW) parameters for the zinc ion from Li et al. (Li et al., 2013).

Production simulations were performed with the GPU‐accelerated pmemd engine in AMBER 2018 (Case et al., 2018). Simulations of a single copy of each protein in water and KCl were performed at two concentrations: *b*_KCl_ =0.15 mol kg^−1^ and *b*_KCl_ =2 mol kg^−1^. The low concentration is typical of the cytoplasm of mesophilic organisms. The high concentration was selected because the highest for which our chosen force fields still reliably reproduce electrolyte structure as measured through the solute activity (Joung & Cheatham, 2008). The simulation boxes were cubic with approximate edge lengths of 100 Å, maintaining a minimum distance of around 20 Å between the protein surface and the box face. The composition of each simulated system is shown in Table S1. Periodic boundary conditions were applied in all dimensions. Nonbonded interactions were cutoff at 12 Å, with electrostatic interactions calculated using the Particle Mesh Ewald method for interparticle distances beyond that cutoff. The SHAKE algorithm (Ryckaert et al., 1977) constrained most bonds involving the hydrogen atom, which enabled using a 2 fs time step for most simulated systems. The only exception occurred in the simulations involving carbonic anhydrase proteins because the SHAKE algorithm could not be applied to the water molecule coordinating with the zinc metal center; in this case, a 1 fs time step was employed. Temperature control was achieved using a Langevin thermostat with a collision frequency of 0.01 ps^−1^, maintaining an average system temperature of 298 K. Production simulations ran under the NVT ensemble, extending for 0.5 μs for carbonic anhydrase and 1 μs for other proteins, with configurations saved every 100 ps for subsequent analysis. The same simulations were already analyzed to understand the composition and dynamics of the protein solvation shells, as reported in reference (Geraili Daronkola & Vila Verde, 2021), and to understand the impact of salt on the dynamics of the protein, as reported in reference (Geraili Daronkola et al., 2024). Here, we focus exclusively on the structure and energetics of the hydrogen bond network of protein solvation shells.

#### 2.2.1 Characterizing the geometry of hydrogen bonds

We calculated distributions, $p\left(d,\cos\left(\theta \right)\right)$, of the distance, d, between the donor and acceptor heavy atoms, and of the hydrogen‐centered angle, θ, formed by the 3 atoms, by sampling from the 10,000 saved production run configurations for each simulated system. We considered water–water hydrogen bonds accepted by water oxygen atoms within 3.5 Å of a carbon or oxygen protein atom, as well water‐to‐protein hydrogen bonds, where the acceptor is a protein oxygen or nitrogen atom and the water oxygen atom is a maximum of 3.5 Å away from the acceptor. The water‐to‐carboxylate distribution was obtained by considering water molecules with the oxygen atom within 3.5 Å of a carboxylate oxygen belonging to an aspartate or glutamate side chain. The hydrogen bond network of the solvent in the bulk was characterized by an equivalent distribution function ($p_{b,\text{bulk}}$) at each salt concentration b, obtained by considering all water oxygen acceptor atoms more than 10 Å of all carbon or oxygen protein atoms of protein 2KAC and sampling every 100th saved configurations. These two reference distributions were calculated only from the simulations of protein 2KAC and then used for all the proteins because the solvents (with molality *b*_KCl_ =2 mol kg^−1^ and *b*_KCl_ =0.15 mol kg^−1^) were the same for all proteins. The simulations were analyzed through Tcl/Tk scripts ran within the VMD (Humphrey et al., 1996) software package, and the data was subsequently binned using python scripts.

## 3 RESULTS AND DISCUSSION

### 3.1 Solvation shell spectroscopy

**FIGURE 1:**
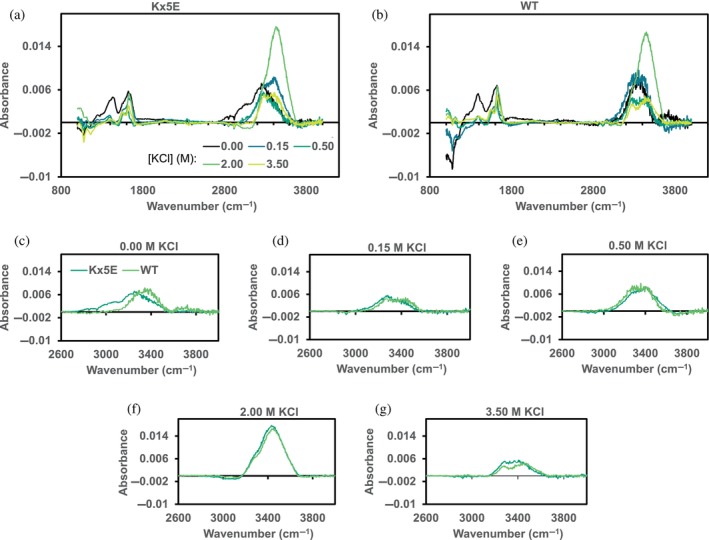
IR solvation shell spectra of (a) halophilic protein L (Kx5E) and (b) its mesophilic (WT) version as a function of KCl concentration. (c–g) Solvation shell spectra of the two proteins at each KCl concentration. Panels (a) and (b) have the same color scale; panels (c–g) have the same color scale.

The solvation‐shell spectra of the halophilic protein L (Kx5E) and its mesophilic (WT) version as a function of KCl concentration is shown in Figure 1. Here, panels (a) and (b) compare the spectra of Kx5E and WT, respectively, as a function of salt concentration, while panels (c‐g) compare the solvation shell spectra of Kx5E and WT at each of the salt concentrations.

The solvation shell spectra contain the vibrations of both the proteins as well as the water in the solvation shell. The protein amide vibrations are visible at low frequencies in the range [1200, 1700] cm^−1^, while the high frequency region [2600, 3800] cm^−1^ is dominated by the water OH vibrations. The amide vibrations are sensitive to the secondary structure of the protein (Arrondo et al., 1993; Susi & Byler, 1986). The amide I vibration is overlapped with the water OH bend vibration, but the amide II and III bands are unobstructed by the solvent. That the amide vibrations do not change significantly with salt concentration for both the halophilic and mesophilic proteins indicates that both proteins are not denatured by the salt and remain in their native state, as confirmed by CD spectroscopy (Section S1).

The water OH stretch vibration is very sensitive to the local hydrogen‐bonding environment (Corcelli & Skinner, 2005; Loparo et al., 2007; Ojha et al., 2018). Non‐hydrogen‐bonded OH, also called free‐OH or dangling OH bonds, are observed as a relative sharp resonance around 3700 cm^−1^. Hydrogen bonds weaken the OH bond, which cause a shift to lower frequencies and a broadening of the vibration increasing with the strength of the hydrogen bond. As such, the OH spectrum of water in the solvation shell provides a map of the distribution of hydrogen‐bond strengths of water in the solvation shell (Corcelli & Skinner, 2005; Loparo et al., 2007; Nakamoto et al., 1955; Ojha et al., 2018).

Except for perhaps the 0 M KCl solution, the solvation shell spectra of Kx5E and WT are very similar. The variation between Kx5E and WT at each salt concentration is smaller than the variation between the different salt concentrations, which reflects the reproducibility of the experiments at these low protein concentrations. Our previous investigations of proteins were performed at concentrations of 10 mg/mL or higher (Sun & Petersen, 2017). The present experiments were done at 0.5 mg/mL, which pushes the limits of the method. Moreover, for the IR‐SSS experiments with both variants of protein L, it was not possible to have identical KCl and buffer concentrations in the protein solutions and in their respective background solutions, as discussed in the Methods section. Such minor differences in concentration can affect the solvation shell spectra because the OH stretch of water changes with salt concentration. These concentration differences potentially explain part of the differences between the spectra of the same protein at different salt concentrations. We have, however, used the same background solutions for both variants of protein L at each concentration, making comparisons between them at each salt concentration (Figure 1c‐g) more reliable. The SSS with a KCl concentration of 2 M seems significantly different than for the other concentrations, but nearly identical SSS spectra were obtained for WT and Kx5E. Accordingly, we do not see a significant difference in the solvation shell between the WT and Kx5E versions of protein L.

A second data set of the halophilic and mesophilic proteins is shown in the supplementary information (Figure S2) and show qualitatively the same behavior: the difference between Kx5E and WT is smaller than the reproducibility of the experimental method, defined as differences between experimental runs (on different days and/or using slightly different sample preparations). For a given experimental run, the wild‐type and mutant protein IR‐SSS at a given salt concentration are quite similar. We can thus comfortably say that the level of hydration of halophilic and mesophilic proteins is not substantially different, in agreement with the simulations described below and in contrast with the interpretation of ultracentrifugation and neutron scattering experiments (Calmettes et al., 1987; Madern et al., 2000; Pundak & Eisenberg, 1981).

As a control of the method, we also obtained two independent datasets of solvation shell spectra of lysozyme, a mesophilic protein, as a function of the salt concentration. In this experiment, it was possible to ensure that the protein and background solutions had identical KCl and buffer concentrations, as described in the methods section. The lysozyme results are shown in the supplementary information (Figure S3). Here, some variation in both the amide and water OH vibrations as a function of salt concentration are observed, indicating some degree of denaturation and associated changes in the hydrogen‐bond interactions in the solvation shell. Nevertheless, differences in the water OH stretch region as a function of salt concentration are smaller than the uncertainty of the method, suggesting that the solvation shell is not dramatically sensitive to changes in electrolyte concentration.

### 3.2 Simulation

**FIGURE 2:**
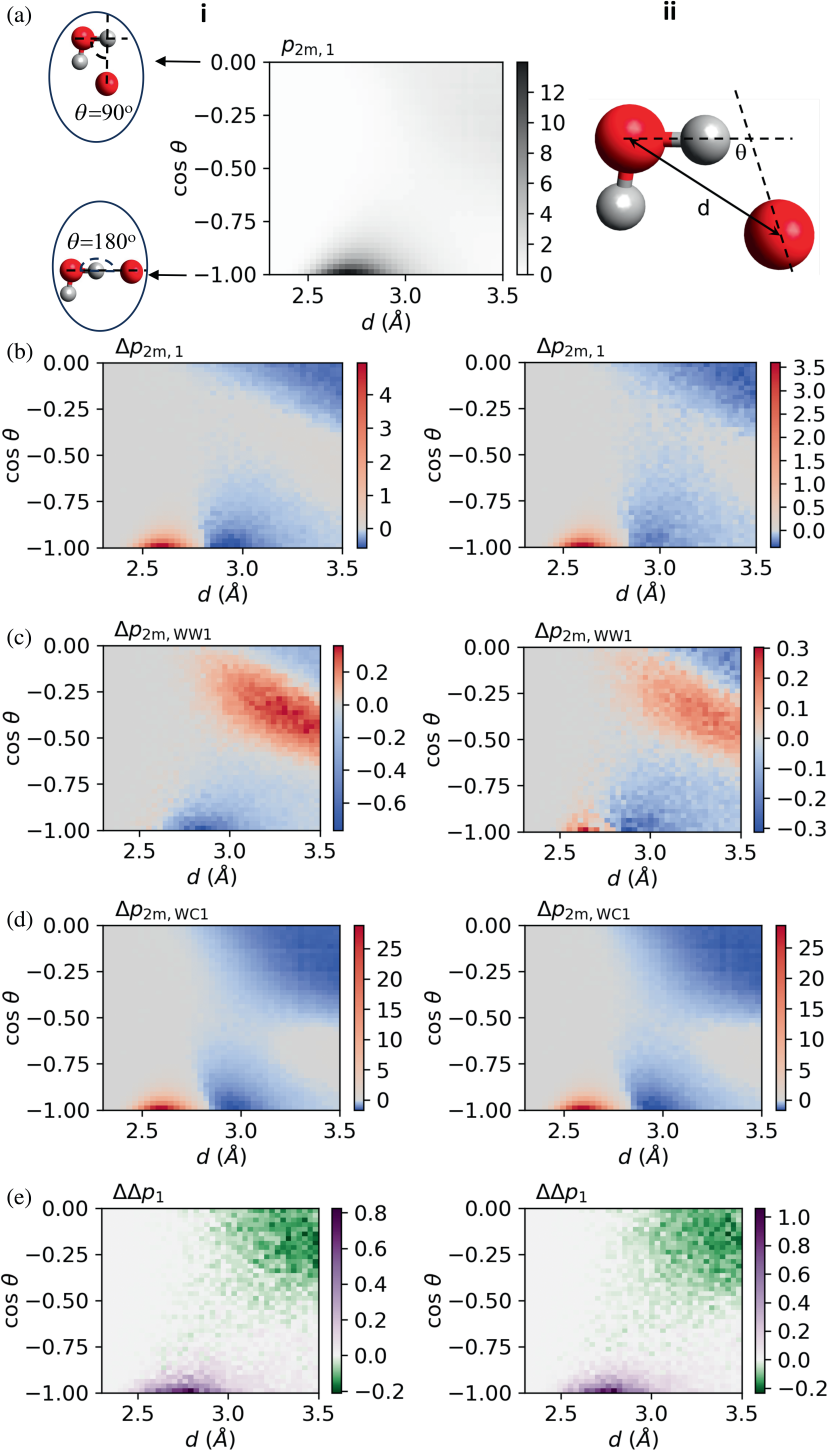
Impact of salt concentration and protein halophilicity on the hydrogen bond network formed by water acceptors in the first solvation shell of (i) halophilic and (ii) mesophilic protein L. (a) Example probability density, $p_{2m,1}\left(d,\cos\;\theta \right)$ considering water–water and water‐to‐carboxylate hydrogen bonds in the solvation shell of the halophilic protein L in $b_{\mathrm{KCl}}=2$ mol kg^−1^. (b) Perturbation of the solvation shell relative to the solvent in the bulk, as measured by $\Delta p_{2m,1}=p_{2m,1}-p_{2m,\text{bulk}}$ (Equation 1). (c) Perturbation of the water–water hydrogen bonds relative to the solvent in the bulk, as measured by $\Delta p_{2m,\mathrm{WW}1}=p_{2m,\mathrm{WW}1}-p_{2m,\text{bulk}}$ (Equation 1). (d) Perturbation of the water‐ carboxylate hydrogen bonds relative to the solvent in the bulk, as measured by $\Delta p_{2m,\mathrm{WC}1}=p_{2m,\mathrm{WC}1}-p_{2m,\text{bulk}}$ (Equation 1). (e) Difference of the perturbation of the solvation shell between two KCl concentrations, as measured by $\Delta \Delta p_{1}=\Delta p_{2m,1}-\Delta p_{0.15m,1}$ (Equation 2). In (b–e), the color bar indicates the magnitude of the difference (or difference of differences) of the indicated probability densities.

We characterize the hydrogen bond network of protein solvation shells through the probability density, $p\left(d,\cos\theta \right)$, of distance, d, and angle, θ, that characterize hydrogen bond geometry. Figure 2a.(i) shows an illustrative distribution, $p_{2m,1}$, obtained for protein 2KAC at *b*_KCl_ =2 mol kg^−1^, and which includes all water–water hydrogen bonds with water acceptors in the first solvation shell as well as water‐to‐protein hydrogen bonds. It shows a maximum in intensity at d≈2.7 Å and cosθ=−1, that is, when the angle $\theta =180^{{}^{\circ}}$, which indicates that the 3 atoms defining the hydrogen bond are perfectly aligned; this geometry has the energetically most favorable hydrogen bonds. Configurations with cosθ approaching zero are only weakly interacting, and therefore not true hydrogen bonds. Nevertheless, in what follows, we refer to the entire population of configurations represented in this plot as a “hydrogen bond” population, as short‐hand for geometric configurations between neighbors.

To understand how the hydrogen bond network of protein solvation shells differs from that of the bulk solvent, we calculated probability density functions ($p_{b,\text{bulk}}\left(d,\cos\theta \right)$) for water–water hydrogen bonds in the bulk at both salt concentrations. In what follows, the distributions will be denoted without explicitly referencing the distribution variables d and cosθ, to simplify notation. Subtracting this distribution from the distribution for a given subpopulation, x, of hydrogen bonds in solvation shells at a given molality b of KCl yields the difference

(1) $$\Delta p_{b,x}=p_{b,x}-p_{b,\text{bulk}},$$

which reveals how the geometry of hydrogen bonds in protein solvation shells differs from that in the bulk solvent. The difference $\Delta p_{b,x}$ is positive for $\left(d,\cos\theta \right)$ configurations more frequent in the solvation shell and negative for those less frequent in the solvation shell relative to the solvent in the bulk. Figure 2b(i,ii) shows this difference for the halophilic and mesophilic protein L for *b*_KCl_ =2 mol kg^−1^; analogous distributions are shown in Figure S4 for all proteins and for both salt concentrations. For all proteins and all salt concentrations, very strong hydrogen bonds (with d<2.8 Å; in red in this figure) are substantially more frequent in the first solvation shell as compared to the bulk, and moderately strong hydrogen bonds (for d>2.8 Å and −1<cosθ<−0.75; in blue) are somewhat less frequent in the first solvation shell. Systematic differences between halophilic and mesophilic proteins exist, but are very small: Very strong hydrogen bonds (with d<2.8 Å) are always systematically slightly more frequent for the halophilic proteins, whereas moderately strong hydrogen bonds (for d>2.8 Å and −1<cosθ<−0.75) are slightly more depleted for those proteins.

We first examine what hydrogen bond populations—water–water or water‐to‐carboxylate—are responsible for these small differences between halophilic and mesophilic proteins. Figure 2c(i,ii) shows the perturbation of water–water hydrogen bonds in the first solvation shell of protein L relative to the bulk solvent, as measured by equation 1 and considering only the subpopulation, WW1, of water–water hydrogen bonds with water acceptors in the first solvation shell of the proteins. Equivalent results for all proteins are shown in Figure S5. We find that strong water–water hydrogen bonds (2.7< *d*/(Å) <2.9) are primarily depleted in the first solvation shell of both types of proteins (blue area in Figure 2c), although the solvation shells of some proteins are also enriched in even stronger water–water hydrogen bonds (2.5< *d*/(Å) <2.7; in red) relative to the bulk solvent. These results show that some of the changes observed in the solvation shell of the proteins relative to the solvent in the bulk indeed originate from changes in water–water hydrogen bonds. Nevertheless, changes in water–water hydrogen bonds do not fully explain the differences between protein solvation shells and the solvent in the bulk.

Halophilic proteins have, on average, more acidic amino acids than mesophilic ones, and carboxylates can accept very strong hydrogen bonds (Kuntz, 1971b). To quantify their impact on protein solvation shells, we apply Equation 1 to the subpopulation, WC1, of water‐to‐carboxylate hydrogen bonds around each protein. Figures 2d(i,ii) and S6 confirm that this subpopulation is primarily responsible for the higher probability of finding very strong hydrogen bonds in solvation shells (in red in Figure 2b,d) relative to the bulk, as intuitively expected.

The question then arises: Should these differences result in systematic differences between the solvation shell spectra of halophilic and mesophilic proteins? To answer it, we mapped the distance and angle distributions obtained in molecular dynamics simulations to vibrational frequency, using as basis experimentally determined OH stretch frequencies as a function of OO distance (Nakamoto et al., 1955). These results, presented in Figures S8 and S9, indicate that halophilic and mesophilic proteins have very similar solvation shell spectra despite their different amino acid composition, in accordance with our experimentally measured spectra.

Finally, we examine if these small differences between the solvation shells of halophilic and mesophilic proteins could translate into different robustness of the hydrogen bond network of their solvation shells to changes in salt concentration. We quantify the robustness of solvation shells by comparing the difference in hydrogen bond geometry relative to the solvent in the bulk at 2 and 0.15 mol kg^−1^ KCl:

(2) $$\Delta \Delta p_{x}=\Delta p_{2m,x}-\Delta p_{0.15,x},$$

where x identifies a given hydrogen bond population in the protein solvation shell. $\Delta \Delta p_{x}$ is positive when the hydrogen bond geometry difference between the solvation shell and the bulk solvent is larger at the higher KCl concentration (*b*_KCl_ =2 mol kg^−1^), negative when it is larger at the lower KCl concentration (*b*_KCl_ =0.15 mol kg^−1^), and zero when it is the same at both concentrations of KCl. In Figure 2e, this difference is shown for halophilic and mesophilic protein L; the same differences are shown in Figure S7 for all proteins. Figures S5 and S6 show analogous quantities calculated for the WW1 and WC1 subpopulations. We find that the $\Delta \Delta p_{x}$ distributions are almost identical between halophilic and mesophilic proteins, indicating that the hydrogen bond network in their solvation shells are equally robust to changes in salt concentration. For both types of protein, high KCl concentrations induce frequent strong hydrogen bonds in the solvation shell (purple region in Figure 2d), at the expense of non‐hydrogen‐bonded configurations (green region in Figure 2d).

## 4 CONCLUDING REMARKS

Prior simulation work by some of us indicates that the concentration of potassium ions and of water in the solvation shell of proteins increases approximately linearly with their surface density in acidic amino acids (Geraili Daronkola & Vila Verde, 2021); the concentration of ions also depends on the bulk concentration of KCl. For both types of proteins and both salt concentrations, the solvation shell is enriched in water and ions relative to the solvent in the bulk (Geraili Daronkola & Vila Verde, 2021). The present results add to this picture in that they indicate that there are no large differences between the hydrogen bond network in the solvation shells of halophilic and mesophilic proteins, despite their different amino acid composition. Small systematic differences were detected in simulation, below the detection limit of IR‐solvation shell spectroscopy. Importantly, changes in hydrogen bond network in protein solvation shells as a function of KCl concentration are very similar for halophilic and mesophilic proteins. The previously advanced scenario that halophilic proteins are enriched in acidic amino acids to compete with ions in solution for available water is thus not supported, given that both the water content (Geraili Daronkola & Vila Verde, 2021) and the hydrogen bond network in protein solvation shells are equally robust to changes in KCl concentration for halophilic and mesophilic proteins. We note that, while the WT and mutant protein L used in the IR‐SSS experiments differ only in their content in acidic amino acids, the halophilic–mesophilic protein pairs investigated in simulations have other compositional differences that also exist between natural halophilic and mesophilic proteins. The simulations indicate that our conclusions should hold for halophilic–mesophilic protein pairs with a wide range of compositional differences, and suggest specific systems that could be investigated experimentally in the future.

Cells have a typical protein density of 200 mg/mL (Milo, 2013), so proteins must remain soluble at these high concentrations. Solubility measurements of 3 variants of protein L—halophilic, halotolerant, and mesophilic—by some of us (Tadeo et al., 2009) and of multiple mesophilic proteins by others (Dumetz et al., 2007; Guo et al., 1999; Tessier & Lenhoff, 2003) indicate that the solubility of some proteins decreases substantially at high salt concentration. It seems possible that excess acidic amino acids in acidic proteomes is evolutionary driven by the fact that they are better than positively charged ones at maintaining sufficient protein solubility to be compatible with function and thus life at high salt concentrations. They might ensure solubility, if not by maintaining long range protein repulsion (beyond 0.5 M electrostatic screening is complete (Guo et al., 1999)), then by the combined effects of having particularly favorable electrostatic interactions with the solvent (Geraili Daronkola & Vila Verde, 2023; Loche et al., 2018) and having reduced hydrophobic solvent accessible surface area as compared with positively charged amino acids. We note, however, that the excess acidic amino acids typically present in halophilic proteins appears to be larger than the number necessary to achieve this effect. For example, the macroscopic surface tension of water is 72 mN m^−1^; for aqueous KCl solutions at b=2 mol kg^−1^, it is 76 mN m^−1^ (Ali et al., 2009). Noting that the microscopic surface tension of water is approximately half of the macroscopic one (Date & Dominy, 2013; Sharp et al., 1991), the extra cost of moving a typical protein with 10,000 Å hydrophobic solvent accessible surface area (Geraili Daronkola et al., 2024) from pure water to that salt solution is $10000\times \left(76-72\right)\times 0.5=120$ kJ mol^−1^. This cost is more than compensated by the introduction of a single acidic amino acid. (Geraili Daronkola & Vila Verde, 2023) In view of these considerations, it is clear that further work is necessary to ascertain the role of the number and distribution of acidic amino acids in the maintenance of protein solubility at high KCl concentration. Importantly, the impact of protein composition and of electrolyte concentration on the local dielectric constant of solvation shells should be explicitly investigated, because noticeable changes in dielectric constants—potentially impacting protein aggregation—can result from fairly small changes in solvent structure.

Prior experimental work by some of us indicates that thermodynamic stability of halophilic proteins—but not mesophilic ones—is very sensitive to KCl concentration and pH (Herrero‐Alfonso et al., 2024), halophilic proteins being equally or even more stable than mesophilic ones at high salt concentrations. Those results suggest that a high content in acidic amino acids is *well tolerated* at high salt concentration, but is perhaps *not necessary* to have sufficient thermodynamic stability under those conditions, although potentially being necessary to ensure sufficient solubility. In contrast, at low KCl concentrations, a high content in acidic amino acids is typically not well tolerated (Herrero‐Alfonso et al., 2024) since it decreases stability at low salt concentration because of electrostatic repulsion, and likely also because of changes in the preferential ion exclusion between folded and unfolded states given the reduced hydrophobic solvent accessible surface area of halophilic proteins relative to mesophilic ones (Herrero‐Alfonso et al., 2024; Tadeo et al., 2009). These trends, inferred from studies of a limited number of proteins, will be further investigated by some of us using simulations with a larger protein dataset, building up on the fact that, for mesophilic proteins, the influence of salt on protein stability can be well captured by simple models that include Poisson–Boltzmann electrostatics and the hydrophobic effect (Date & Dominy, 2013), and which we will further refine to include insight into the local dielectric constant near halophilic and mesophilic proteins.

In light of the findings presented here and the discussion above, it is important to consider that halophilic environments have been proposed as possible sites of prebiotic proteogenesis (Dundas, 1998; Rode, 1999), because amino acids characteristic of halophilic proteins are abundant in the prebiotic amino acid set, and proteins composed of those amino acids can fold (Longo et al., 2013; Longo & Blaber, 2014). Given this possibility, it remains unclear whether halophilic proteomes evolved from mesophilic ones to accommodate high salt conditions, or if (some) halophilic proteomes were pressured to become less acidic because halophilic proteins could not be stable at low salt concentration, and no longer needed the acidic proteome to remain soluble.

## AUTHOR CONTRIBUTIONS

**H. Geraili Daronkola:** Investigation; writing – original draft; visualization; methodology; validation; formal analysis. **Bashar Moussa:** Investigation; methodology; validation. **Óscar Millet:** Investigation; resources. **Oktavian Krenczyk:** Investigation; validation. **Gabriel Ortega‐Quintanilla:** Investigation; writing – review and editing; writing – original draft; resources. **Poul B. Petersen:** Investigation; writing – review and editing; supervision; writing – original draft; data curation; formal analysis; methodology; validation; visualization. **Ana Vila Verde:** Conceptualization; writing – original draft; writing – review and editing; project administration; supervision; funding acquisition; data curation; investigation; formal analysis; visualization.

## Supporting information


**Data S1.** Experimental details and results for CD and DLS experiments, a second data set of IR‐SSS spectra for protein L, IR‐SSS spectra of lysozyme, details of the composition of the simulated systems, simulation results for all simulated proteins.

## Data Availability

Simulation input files, selected simulation output files and scripts to produce published images containing simulation data can be found in https://doi.org/10.17877/RESOLV-2024-m32ubdo9.
