# Supplementary material for: How sensitive are protein hydration shells to electrolyte concentration and protein composition?
Source: Protein Sci. 2024 Dec 14;34(1):e5241. doi: 10.1002/pro.5241 (PMC11645670; doi:10.1002/pro.5241)
Supplement: Supplementary file 1 — Data S1. Experimental details and results for CD and DLS experiments, a second data set of IR‐SSS spectra for protein L, IR‐SSS spectra of lysozyme, details of the composition of the simulated systems, simulation results for all simulated proteins. [file PRO-34-e5241-s001.pdf]

Supplemental information

How sensitive are protein hydration shells to electrolyte concentration and protein composition?

S1 CD and DLS

S1.1 Methods

The lyophilized protein powders were dissolved in double distilled water to reach 1.875 mg/mL protein concentration in solutions with 75 mM sodium phosphate buffer at pH 6 and with {0.15, 0.5, 2, 3.5} M concentration of KCl. To remove potential aggregates or undissolved material, the solutions were centrifuged (2900 g) and the supernatants were passed through 20 nm filters (Cytiva, Whatman). The concentration was determined by a Biospectrometer (Eppendorf) at 280 nm using an absorption coefficient of 9970 $M^{-1}cm^{-1}$. After centrifugation and filtering the protein concentrations were 10 % lower than before. For all KCl concentrations, protein folding was investigated by CD spectroscopy using a Chirascan plus CD Spectrometer (Applied Photophysics). A UV cuvette with 0.5 mm path length was employed and the proteins were diluted to a concentration of 0.3 mM. Further, the extent to which protein association in dimers or larger aggregates occurred was investigated with static and dynamic light scattering (SLS and DLS, respectively). The Dyna Pro Nanostar instrument (Wyatt Technology) served to measure both at the same time.

S1.2 Results

CD spectra of the mesophilic and the halophilic protein L, shown in Fig. S1, did not significantly change as a function of KCl concentration, indicating that proteins do not significantly unfold at the KCl concentrations used in the study.

Furthermore, the results (not shown) obtained from SLS and DLS measurements show that the mesophilic as well as the halophilic protein variant does not change its monomeric appearance over the full range of KCl concentration from 0 to 3.5 M. For all KCl concentrations, DLS data reveal a radius of the protein of (1.30 $\pm$ 0.03) nm for the wilt type (WT) and (1.35 $\pm$ 0.05) nm for the mutant protein L while SLS data lead to a mass of (6.2 $\pm$ 0.4) kDa for the WT and (6.7 $\pm$ 0.9) kDa for the mutant. Both results correspond to monomeric proteins as their mass equals 7.0 kDa; under the assumption of a spherical shape this corresponds to a radius of 1.3 nm is estimated.

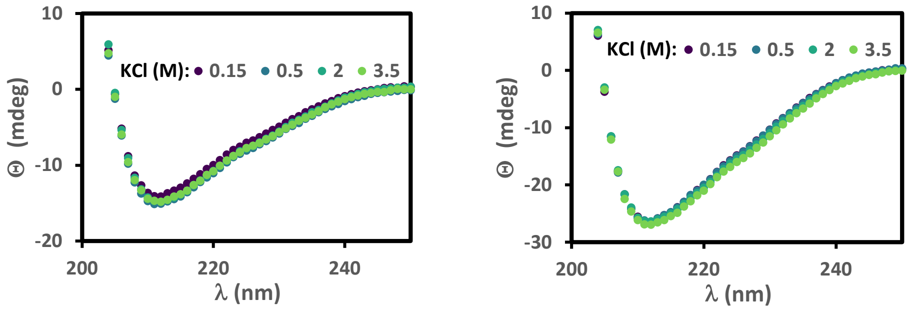

Fig. S1 CD spectrum of wild type (left) and Kx5E (right) protein L, for different KCl concentrations.

S2 Solvation shell spectroscopy

Fig. S2 shows a second dataset for the halophilic and mesophilic protein L, obtained on a separate day and with different solutions as the dataset shown in Fig. 1 in the main text. This data set shows some differences between WT and Kx5E at 2.0 and 3.5 M KCl, while the lower salt concentrations are very similar in agreement with the main data set. Again the 2 M KCl data seems further off than the other samples so overall this data set also supports that there are no major changes in the solvation shell between the WT and Kx5E versions of the protein.

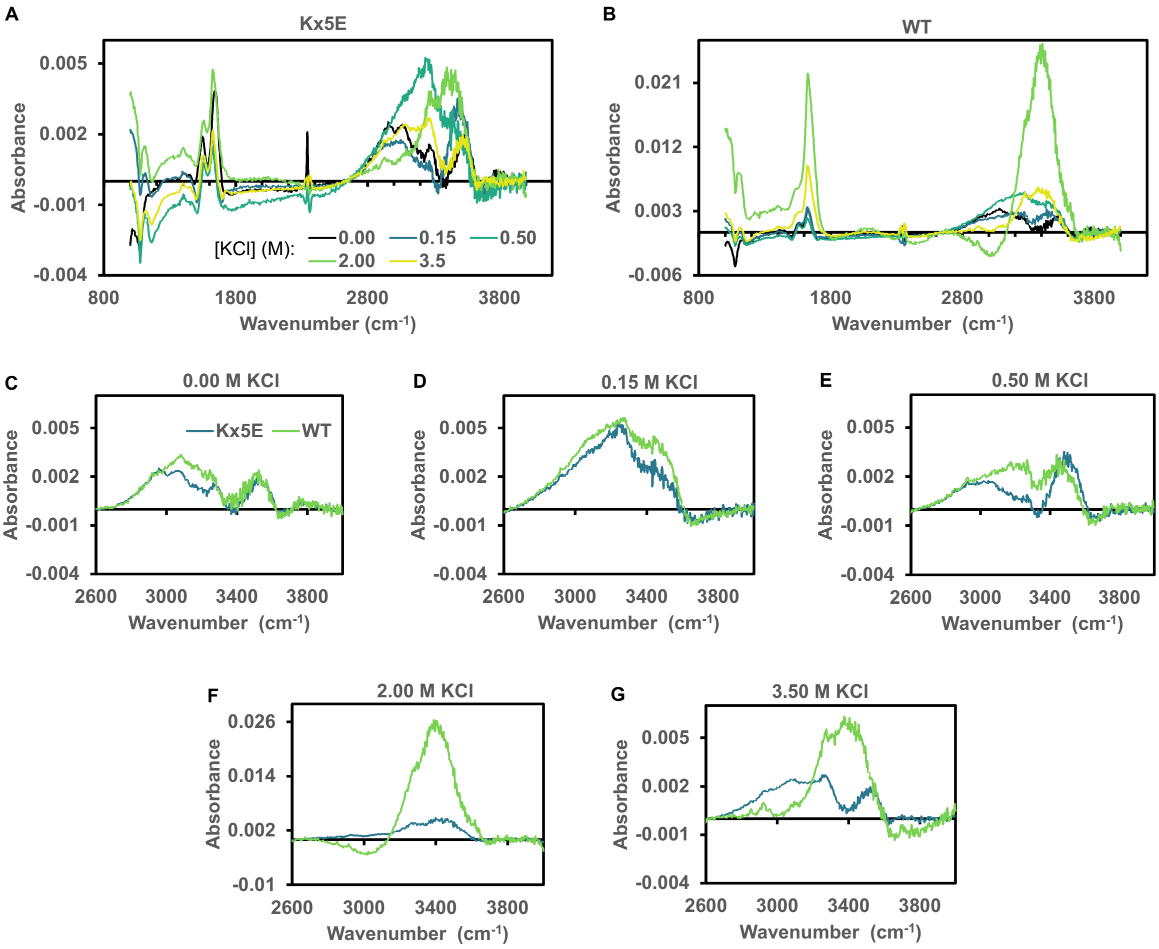

Fig. S2 Second dataset of IR solvation shell spectra of (**A**) halophilic protein L (Kx5E) and (**B**) its mesophilic (WT) version, as a function of KCl concentration. (**C-G**) Solvation shell spectra of the two proteins at each KCl concentration concentration. Panels A and B have the same color scale; panels C-G have the same color scale.

Fig. S3 shows two independent datasets for chicken egg white lysozyme, obtained in separate days and with different solutions. For lysozyme some variation in the amide vibrations is observed with salt concentration, indicating some degree of change in the secondary structure of the protein. However, the data acquired on the different days are quite consistent and, within the uncertainty of the method, substantial changes in the solvation shells between the two data sets are not observed.

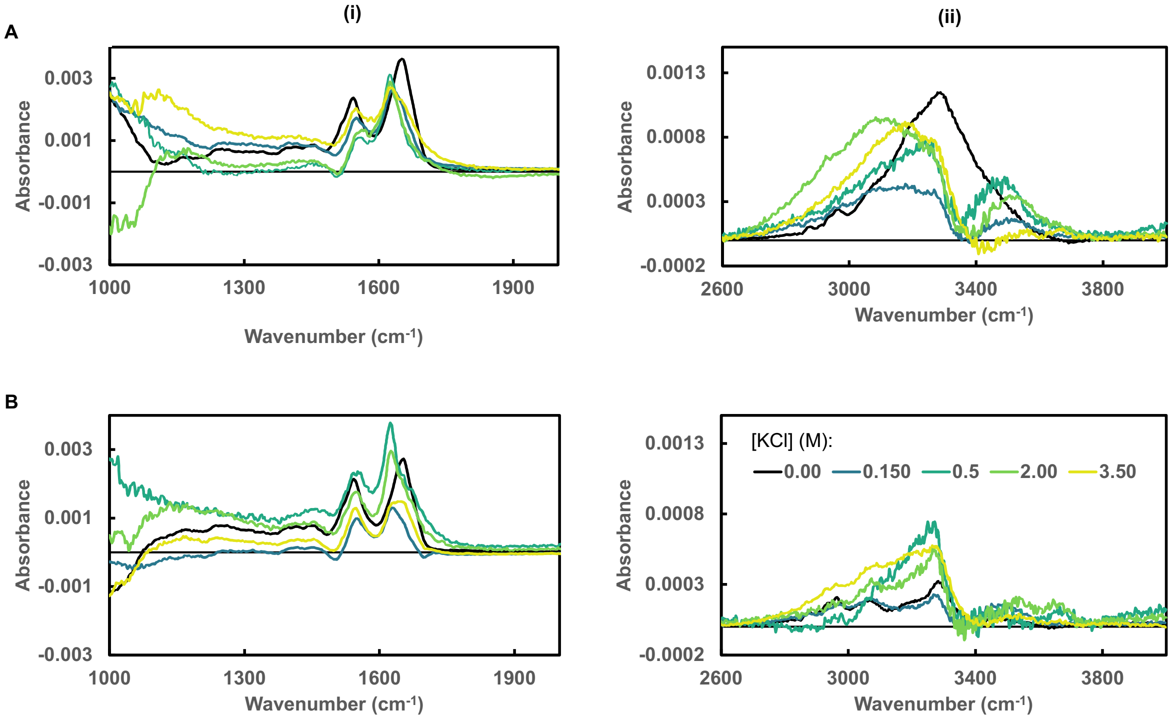

Fig. S3 Solvation shell spectra of lysozyme as a function of KCl concentration. (**A,B**) Two datasets obtained in different days with freshly prepared solutions, as described in section 2.1. (**i**) Zoom in on the 1000 cm^{-1} to 2000 cm^{-1} spectral region, for easier viewing of the amide vibrations; (**ii**) zoom in on the 2600 cm^{-1} to 4000 cm^{-1} spectral region, for easier viewing of the OH stretch band. All panels share the same color scale.

S3 Simulation

S3.1 Composition of the simulated systems.

Table S1 Number of water molecules (n_{WAT}), number of potassium ions (n_{K^+}), and chloride ions (n_{Cl^-}) of the simulated proteins. Each simulation box contained a single copy of each protein.

Halophiles					Mesophiles				
pdb (b_{KCl})		n_{WAT}	n_{K^+}	n_{Cl^-}	pdb (b_{KCl})		n_{WAT}	n_{K^+}	n_{Cl^-}
1DOI	2 mol/kg	39880	1466	1437	1FRD	2 mol/kg	27975	1020	1008
	0.15 mol/kg	50254	165	136		0.15 mol/kg	36496	110	98
2KAC	2 mol/kg	29841	1090	1075	1HZ6	2 mol/kg	33258	1201	1199
	0.15 mol/kg	37005	115	100		0.15 mol/kg	41798	115	113
3RWT	2 mol/kg	60040	2194	2167	1ZKJ	2 mol/kg	48161	1740	1743
	0.15 mol/kg	72261	222	195		0.15 mol/kg	56618	150	153
4CNX	2 mol/kg	39324	1434	1417	1V9E	2 mol/kg	40087	1444	1444
	0.15 mol/kg	47661	146	129		0.15 mol/kg	48344	130	130
2ITH	2 mol/kg	42184	1535	1520	2L28	2 mol/kg	36220	1309	1305
	0.15 mol/kg	51807	155	140		0.15 mol/kg	44775	125	121

S3.2 Structure of the hydrogen bond network in protein solvation shells

The results in Figs. S4, S5, S6 and S7 are discussed in the main text.

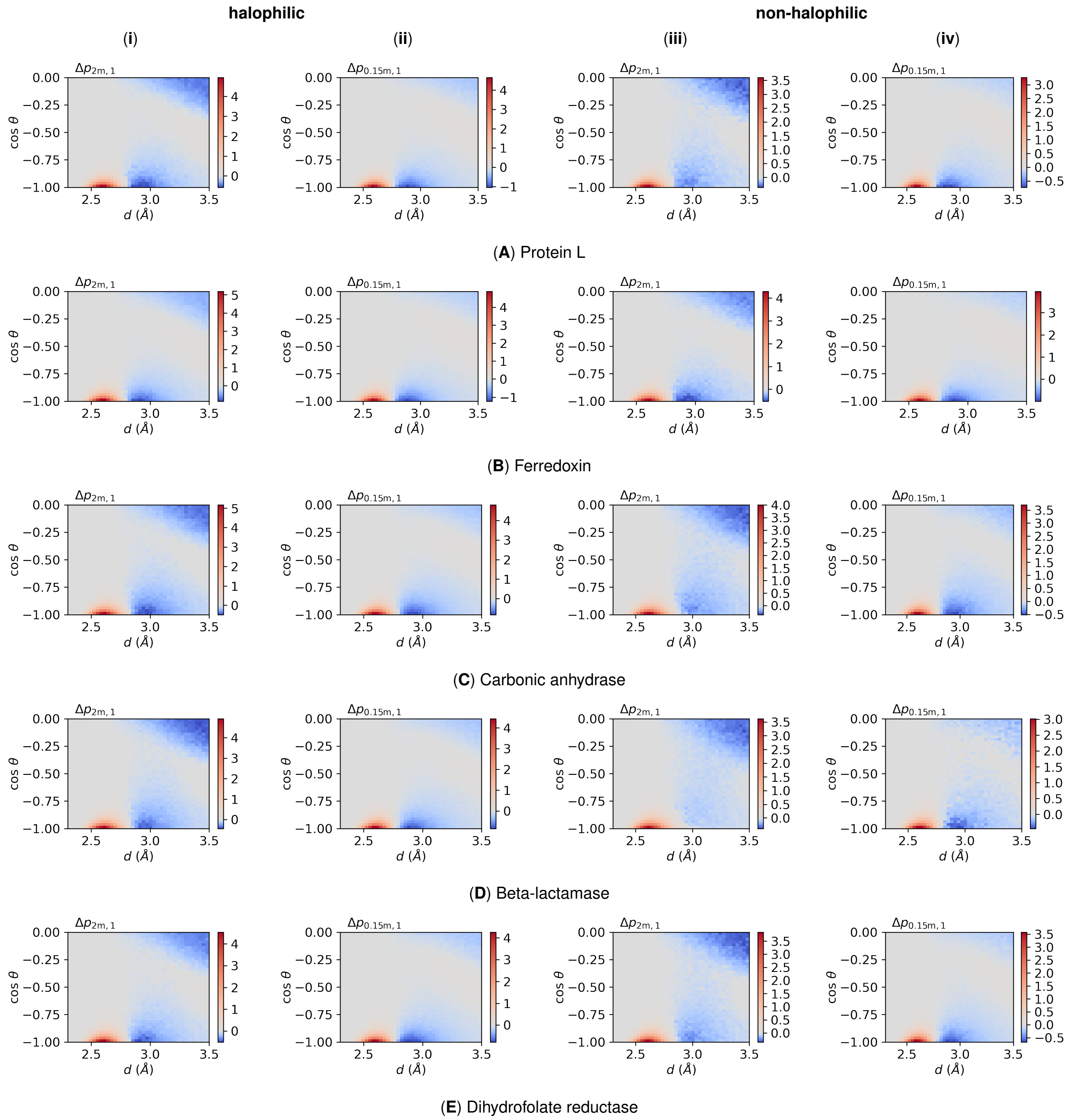

Fig. S4 Solvation shell perturbation relative to the bulk, for the indicated (**i,ii**) halophilic or (**iii,iv**) non-halophilic proteins at low and high KCl concentrations, expressed as $\Delta p_{\mathrm{b},1} = p_{\mathrm{b},1} - p_{\mathrm{b,bulk}}$ where b is the molality of KCl. For all proteins and all salt concentrations, very strong hydrogen bonds (with $d < 2.8$ Å; in red) are substantially enhanced in the first solvation shell as compared to the bulk, but moderately strong hydrogen bonds (for $d > 2.8$ Åand $\cos\theta < -0.75$; in blue) are depleted from the first solvation shell. Very strong hydrogen bonds are always enhanced at the higher salt concentration, whereas moderately strong hydrogen bonds are more depleted at low salt concentrations. Very strong hydrogen bonds are always slightly but systematically enhanced for the halophilic proteins relative to the mesophilic ones, whereas moderately strong hydrogen bonds are slightly more depleted for halophilic proteins.

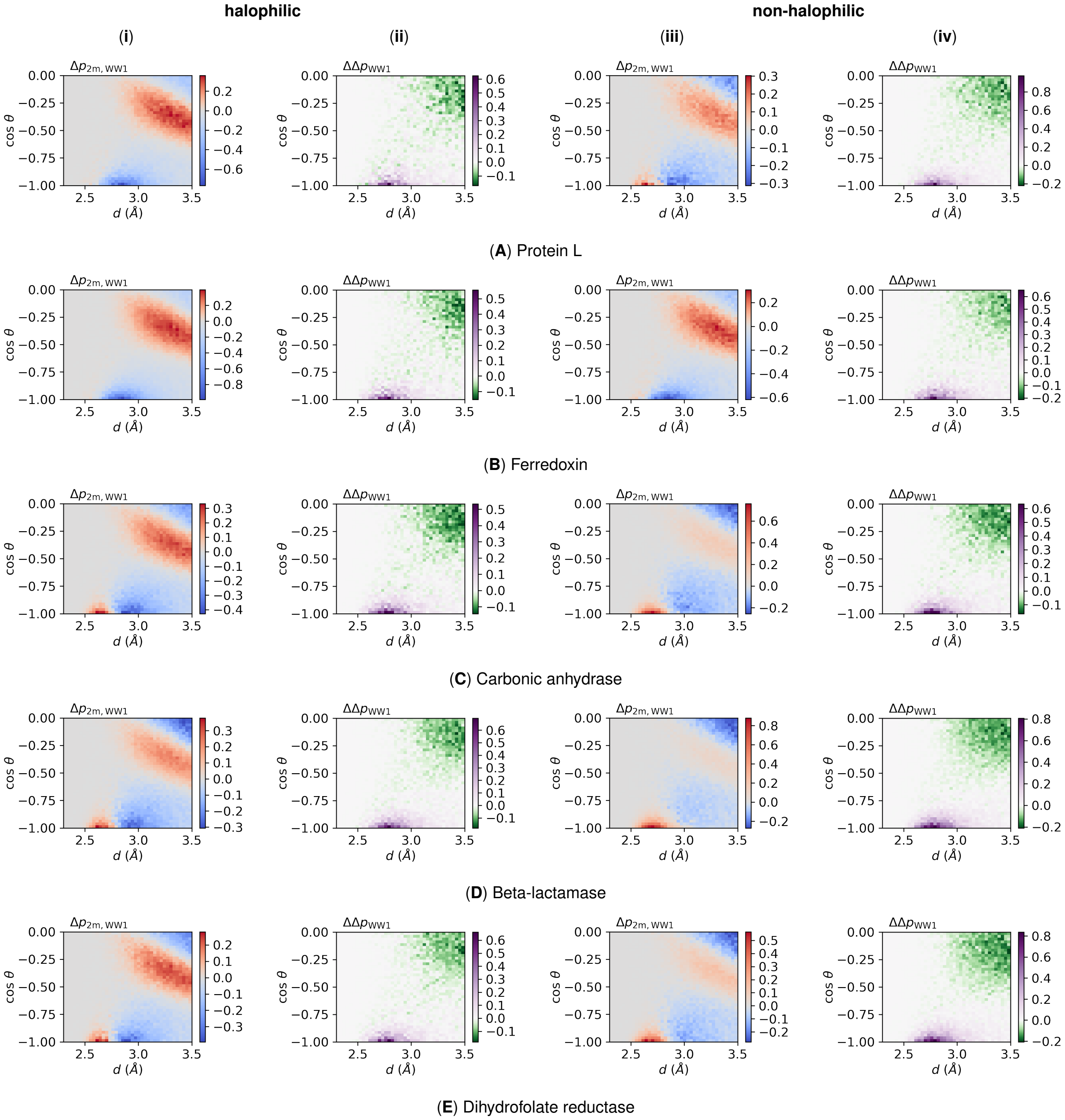

Fig. S5 Impact of salt concentration on water-water hydrogen bonds with donors in the first solvation shell of the indicated (**i,ii**) halophilic and (**iii,iv**) non-halophilic proteins, expressed as (**i,iii**) the perturbation relative to the solvent in the bulk, quantified as $\Delta p_{\mathrm{b,WW1}} = p_{\mathrm{b,WW1}} - p_{\mathrm{b,bulk}}$ for $b = 2$ mol·kg^{-1}, or (**ii,iv**) expressed as the difference in solvation shell perturbation between the low and high concentrations, quantified as $\Delta\Delta p_{\mathrm{WW1}} = \Delta p_{\mathrm{2m,WW1}} - \Delta p_{\mathrm{0.15m,WW1}}$. The distributions in (**i,iii**) show that the water-water hydrogen bond network in the solvation shell is perturbed relative to the bulk: very strong water-water hydrogen bonds are enhanced (red regions for $d < 2.7$ Å)in the solvation shell than in the solvent, at the expense of moderately strong hydrogen bonds (blue regions for $2.7 < d/(\text{Å}) < 3.1$) . The distributions in (**ii,iv**) show that this strengthening (purple areas) is marginally larger at high salt concentration and for the non-halophilic proteins.

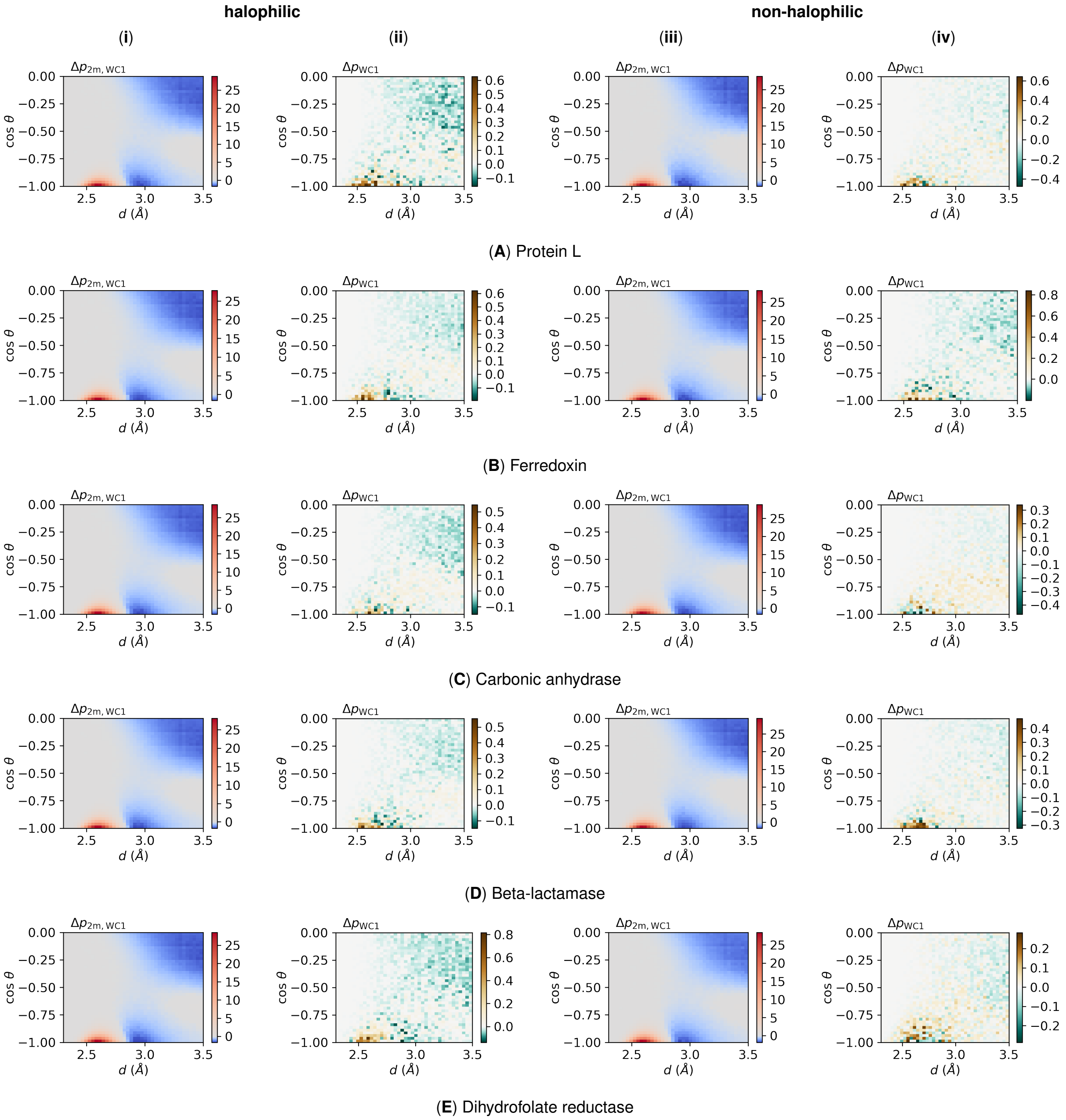

Fig. S6 Impact of salt concentration and protein halophilicity on water-carboxylate hydrogen bonds with water donors in the first solvation shell of (**i,ii**) halophilic or (**iii,iv**) non-halophilic proteins, expressed as (**i,iii**) the difference $\Delta p_{\mathrm{2m,WC1}} = p_{\mathrm{2m,WC1}} - p_{\mathrm{2m,bulk}}$, which quantifies how the distance and angle distribution of water-to-carboxylate (WC) hydrogen bonds differs from that for the solvent in the bulk, for proteins in an aqueous solution with $b_{\mathrm{KCl}} = 2$ mol·kg^{-1}, and (**ii,iv**) as the difference $\Delta p_{\mathrm{WC1}} = p_{\mathrm{2m,WC1}} - p_{\mathrm{0.15m,WC1}}$ of the probability density of observing water-to-carboxylate configurations with the indicated distance and angle at high and low KCl concentration. The distributions in (**i,iii**) show that hydrogen bonds donated by water to carboxylate groups are much stronger than typical hydrogen bonds in aqueous solvent, as indicated by the abundance of hydrogen bonds with $d < 2.7$ Å (red areas) and the decrease in less strong hydrogen bonds ($d > 2.7$ Å, for $\cos\theta < -0.75$; in blue). These results show that water-carboxylate hydrogen bonds contribute substantially to the very strong hydrogen bond peaks in Fig. S4. The distributions in (**ii,iv**) show that the extra population of very strong water-to-carboxylate hydrogen bonds relative to the bulk solvent increases marginally for higher KCl concentration, but to the same extent for halophilic and non-halophilic proteins. These distributions show that differences between high and low salt concentration observed in Fig. S7 (purple areas) do not primarily reflect the contribution of water-to-carboxylate hydrogen bonds.

(**i**) **halophilic** (**ii**) **non-halophilic**

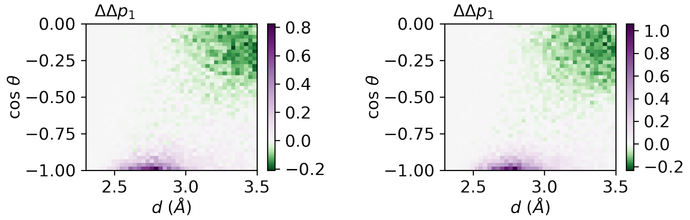

(**A**) Protein L

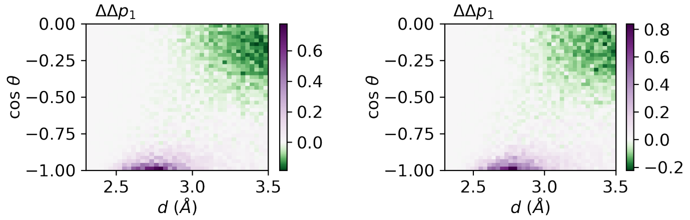

(**B**) Ferredoxin

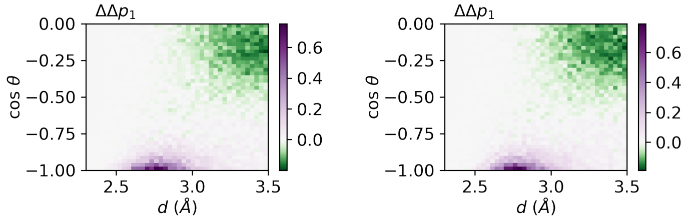

(**C**) Carbonic anhydrase

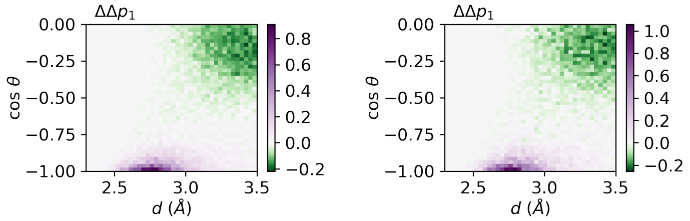

(**D**) Beta-lactamase

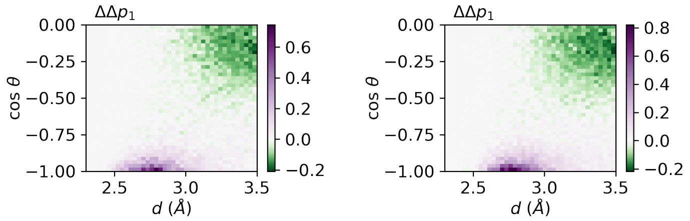

(**E**) Dihydrofolate reductase

Fig. S7 Difference between high and low salt concentration of the solvation shell perturbation relative to the bulk, for the indicated (**i**) halophilic or (**ii**) non-halophilic proteins, expressed as $\Delta\Delta p_1 = \Delta p_{2\text{m},1} - \Delta p_{0.15\text{m},1}$, where $\Delta p_{\text{b},1} = p_{\text{b},1} - p_{\text{b,bulk}}$ quantify the perturbation of the first solvation shell relative to the solvent in the bulk with b KCl molality. The magnitude of the solvation shell perturbation relative to the solvent in the bulk depends on the bulk concentration of KCl. Very strong hydrogen bonds are always enhanced at the higher salt concentration, and are slightly but systematically enhanced for the halophilic proteins relative to the mesophilic ones (purple areas in plots, for $d < 2.8$ Å). This difference does not primarily reflect water-to-carboxylate hydrogen bonds (see Fig. S6), however. Moderately strong hydrogen bonds are less depleted at high salt concentrations (purple areas in plots, but for $d > 2.8$ Å).

S3.3 Estimated solvation shell spectra in protein solvation shells

The relationship between the OH stretch frequency and the distance between donor and acceptor atoms has been experimentally investigated for a wide range of compounds. We took the data from Nakamoto et al.(1) and fitted it with a linear function for OO distances below 2.79336 Å, with an exponential for distances beyond that cutoff. The fits are shown in Fig. S8.

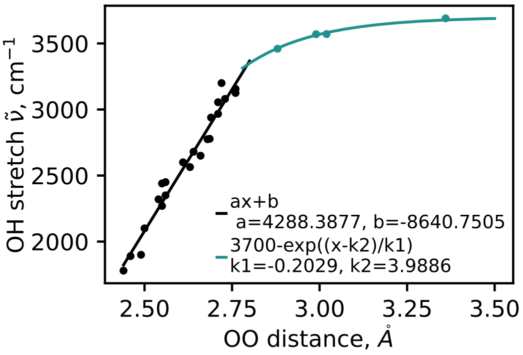

Fig. S8 Fits of the OH stretch frequency as a function of the distance between donor and acceptor oxygens. Data from Nakamoto et al. (1)

We used the functions shown in Fig. S8 to estimate the solvation shell spectra of halophilic and mesophilic proteins from the nearest neighbor distance distribution of the solvation shell. This distribution was obtained from the distribution of OO distances and angles in the solvation shell of each protein (e.g., Fig.2A.i in the main text) by including only distance-angle combinations corresponding to nearest neighbors (those for which the criterium $\cos\theta < (d/(\AA) - 3.5) **2 - 1$ was met). This procedure yields a NN distribution for pure water that agrees well with that obtained using x-ray Raman based extended x-ray absorption fine structure (EXAFS) spectroscopy (2). The NN distribution for the solvation shell of halophilic protein L, as well as the calculated spectra of halophilic and mesophilic protein L are shown in Fig. S9; similar results (not shown) were obtained for other proteins. We note that the calculated spectra look substantially different from the real spectra – in particular, in the lower frequency range between 3000 and 3200 cm^{-1}. This range is dominated by intra- and inter-molecular couplings between the OH vibrations of the water molecules (3, 4), which are not captured in the simple framework used to estimate the spectra. Despite these limitations, these results show that differences in the solvation shell spectra of halophilic and mesophilic proteins are small.

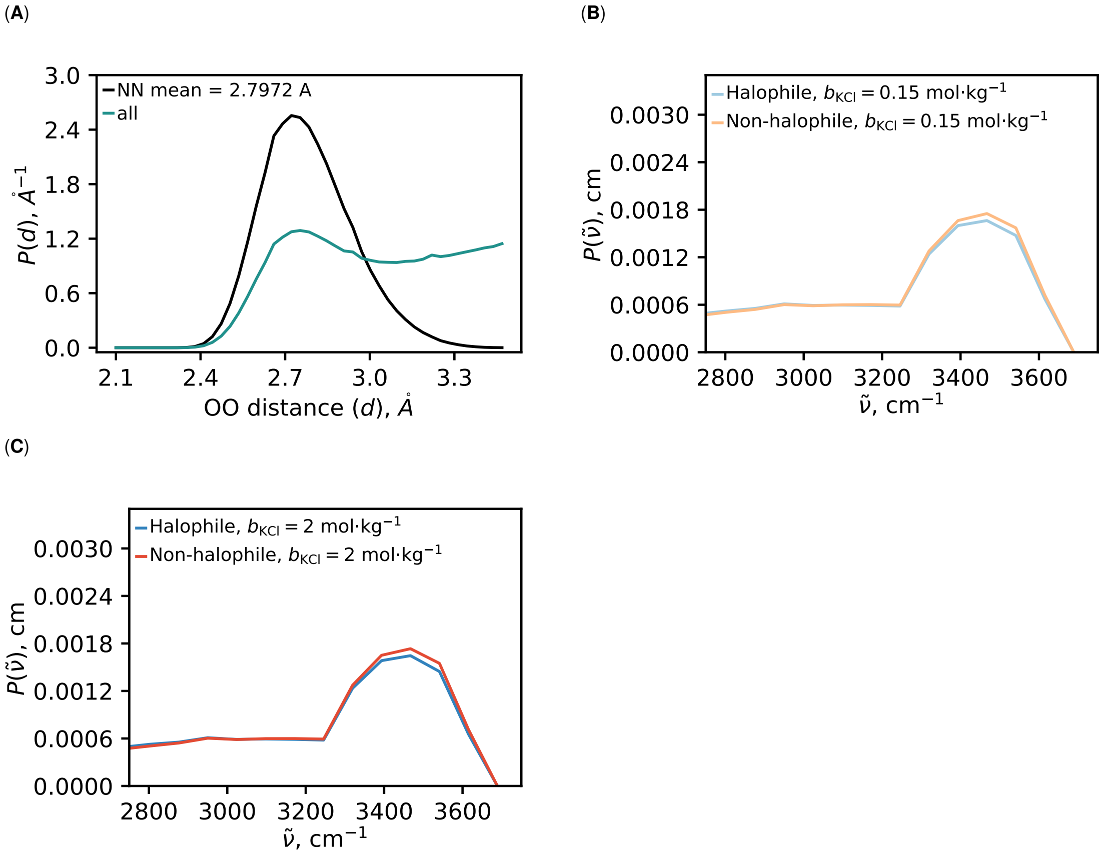

Fig. S9 Estimating the solvation shell spectra of halophilic and mesophilic protein L from nearest neighbor (NN) distances. (**A**) Distribution of distances for hydrogen bonds with oxygen acceptors in the first solvation shell of halophilic protein L, as defined in the main text, considering all oxygens ("all"; from data in Fig. 2A.i) or only nearest neighbors ("NN"; also from data in Fig. 2A.i but with the NN condition described in the text). (**B**) Estimated spectra as a function of wavenumber, $\tilde{\nu}$, computed from the functions displayed in Fig. S8, for halophilic and mesophilic protein L at low salt concentration. (**C**) Estimated spectra, computed from the functions displayed in Fig. S8, for halophilic and mesophilic protein L at high salt concentration.

References

1 K. Nakamoto, M. Margoshes and R. E. Rundle, Stretching frequencies as a function of distances in hydrogen bonds, *J. Am. Chem. Soc.*, 1955, **77**, 6480–6486.

2 U. Bergmann, A. Di Cicco, P. Wernet, E. Principi, P. Glatzel and A. Nilsson, Nearest-neighbor oxygen distances in liquid water and ice observed by x-ray Raman based extended x-ray absorption fine structure, *J. Chem. Phys.*, 2007, **127**, 174504.

3 K. Ramasesha, L. De Marco, A. Mandal and A. Tokmakoff, Water vibrations have strongly mixed intra- and intermolecular character, *Nat. Chem.*, 2013, **5**, 935–940.

4 A. A. Kananenka and J. L. Skinner, Fermi resonance in OH-stretch vibrational spectroscopy of liquid water and the water hexamer, *J. Chem. Phys.*, 2018, **148**, 244107.
